# Loss of Mhc and Neutral Variation in Peary Caribou: Genetic Drift Is Not Mitigated by Balancing Selection or Exacerbated by Mhc Allele Distributions

**DOI:** 10.1371/journal.pone.0036748

**Published:** 2012-05-24

**Authors:** Sabrina S. Taylor, Deborah A. Jenkins, Peter Arcese

**Affiliations:** 1 School of Renewable Natural Resources, Louisiana State University AgCenter, Baton Rouge, Louisiana, United States of America; 2 Department of the Environment, Baffin Region, Government of Nunavut, Pond Inlet, Nunavut, Canada; 3 Centre for Applied Conservation Research, University of British Columbia, Vancouver, British Columbia, Canada; Macquarie University, Australia

## Abstract

Theory and empirical results suggest that the rate of loss of variation at Mhc and neutral microsatellite loci may differ because selection influences Mhc genes, and because a high proportion of rare alleles at Mhc loci may result in high rates of loss via drift. Most published studies compare Mhc and microsatellite variation in various contemporary populations to infer the effects of population size on genetic variation, even though different populations are likely to have different demographic histories that may also affect contemporary genetic variation. We directly compared loss of variation at Mhc and microsatellite loci in Peary caribou by comparing historical and contemporary samples. We observed that similar proportions of genetic variation were lost over time at each type of marker despite strong evidence for selection at Mhc genes. These results suggest that microsatellites can be used to estimate genome-wide levels of variation, but also that adaptive potential is likely to be lost following population bottlenecks. However, gene conversion and recombination at Mhc loci may act to increase variation following bottlenecks.

## Introduction

Low genetic variation can reduce population growth rate and persistence in the wild by limiting the rate at which populations adapt to environmental change or novel pathogens, and by causing inbreeding-related declines in fitness via the expression of recessive deleterious alleles and/or the loss of heterozygote advantage [1]. These risks have made the assessment, management, and restoration of genetic diversity a priority in conservation biology [1], [2].

Genetic variation in wild populations is commonly estimated using neutral microsatellite loci even though it is functional genes that confer fitness and govern trait variation [3], [4]. If genetic variation at microsatellite loci provides an accurate estimate of genetic variation at functional loci, the choice of marker is inconsequential to questions about genetic diversity and fitness in wild populations. However, because neutral loci are only subject to genetic drift whereas functional loci are affected by drift and selection, the effect of population size on genetic diversity at neutral and functional loci is likely to differ. For example, bottlenecks and drift in small populations can result in low genetic variation at all types of loci [5]–[10], but other research suggests that loss of genetic variation at microsatellite and mitochondrial loci can differ from functional genes of the major histocompatibility complex (Mhc), a family of genes that confers resistance to disease [11]–[15]. Theory suggests that the relative resilience of functional as opposed to neutral genetic variation within populations can arise when balancing selection (heterozygote advantage and/or frequency-dependent selection) on Mhc genes acts to maintain high levels of polymorphism (reviews in [16], [17]). Alternatively, Mhc loci may lose more variation than neutral loci because they may have a high proportion of rare alleles that are easily lost via bottlenecks [15]. It is therefore plausible that neutral genetic variation will not provide a precise measure of genome-wide diversity or adaptive potential.

One powerful but rarely used approach to test if estimates of genetic variation at neutral and functional loci are closely correlated is to compare variation at Mhc and microsatellite loci across a population bottleneck. For example, Aguilar et al. (2004) showed that variation at Mhc genes was maintained following a bottleneck that caused all sampled microsatellite loci to drift to fixation in a small population of Channel Island fox (*Urocyon littoralis dickeyi*). Other studies can be more difficult to interpret, for instance when variation at neutral and functional loci is compared in different populations rather than a single population pre- and post-bottleneck [11], [15], [18]–[22]. For example, comparisons between bottlenecked populations of rare species and large populations of common congeners showed that drift reduced genetic variation at both microsatellite and Mhc loci [7], [18]. However, because differences in demographic history (e.g. population size and/or exposure to disease) can also influence genetic patterns [16], [23], direct comparisons of populations prior to and following population declines are preferable. To our knowledge, no one has directly estimated the loss of genetic variation at neutral and functional loci prior to and following a bottleneck to test whether loss of variation differs for Mhc and microsatellite loci.

We used historic and contemporary DNA to directly compare loss of genetic variation at functional and neutral loci before and after a bottleneck large enough to leave a genetic signature in Peary caribou (*Rangifer tarandus pearyi*), an ideal candidate for our goals. Overall, Peary caribou have declined by 70% since the 1960s and have disappeared from some historically occupied areas [24]–[27]. They have been recognized as Threatened since 1979, as endangered since 1991 [28], and in February 2011, they were listed as Endangered under the Canadian Species at Risk Act. Recent reports highlight particular concerns about levels of genetic diversity and risks related to inbreeding and adaptation to environmental challenges in some populations (e.g. Prince of Wales-Somerset) [28]. There is strong evidence that Peary caribou have suffered substantial and recent bottlenecks that are sufficient to cause detectable losses of genetic variation [29], [30]. In this paper, we test the prediction that the loss of genetic variation following a bottleneck would differ at Mhc and microsatellite loci as a consequence of selection and/or drift on each marker.

## Methods

All necessary permits were obtained for the described field studies. These included a Government of Nunavut, Department of Environment, Wildlife Research Permit (WL 2006-000759) and a Parks Canada Permit (QNP-2006-517).

### Study Population

**Figure 1:**
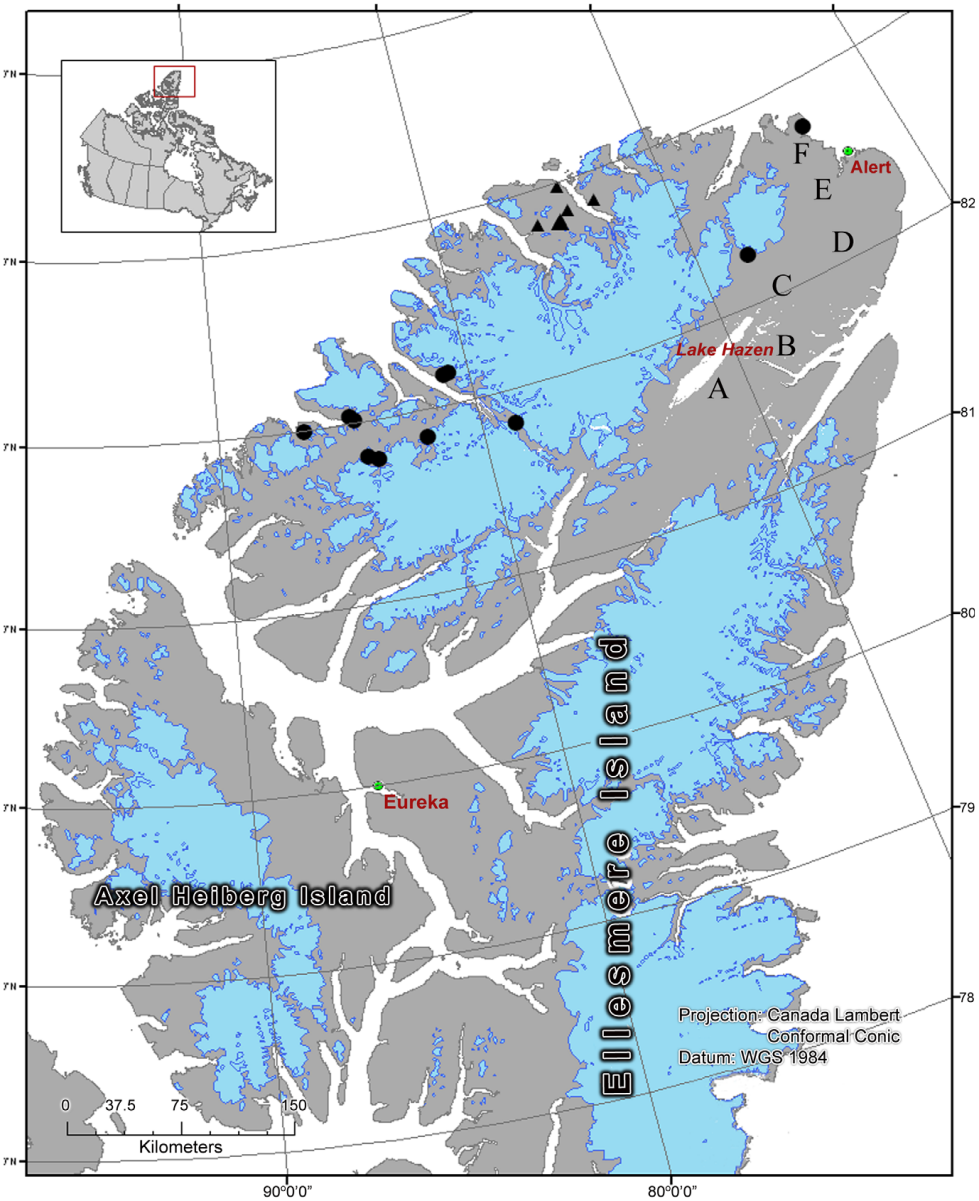
Peary caribou sampling locations. All solid symbols indicate contemporary samples; triangles denote samples that were removed from the analysis to test for a Wahlund Effect; circles denote all other contemporary samples. Multiple contemporary samples were collected from each location. Letters indicate the approximate location of historical samples: A - Lake Hazen (n = 17), B - East of Lake Hazen (n = 1), C - NE of Lake Hazen (n = 7), D - South of Black Cliffs Bay (n = 3), E - Black Cliffs Bay (n = 10), and F - Porter Bay (n = 12).

The Peary caribou is a high Arctic subspecies endemic to Canada and found on the Queen Elizabeth Islands (including Ellesmere Island), Banks and northwest Victoria Islands, Prince of Wales and Somerset Islands, and the Boothia Peninsula [28]. At the turn of the century, explorer Robert Peary collected hundreds of Peary caribou skulls and skins from northern Ellesmere Island, which he deposited at the American Museum of Natural History [31], [32]. More recently (2006), fecal samples were collected on Ellesmere Island from locations proximal to Robert Peary’s sampling locations, affording an excellent opportunity to examine loss of genetic variation over time. In this analysis, we compare DNA from historic tooth samples collected from Porter Bay, Black Cliffs Bay, and Lake Hazen (1905, 1908, 1909, n = 50; Appendix S1) to contemporary fecal samples (2006, n = 51) collected between Porter Bay and Nansen Sound (Figure 1). While the geographic range of the contemporary samples is greater than that of the historical samples, recolonization in Peary caribou appears to occur between distant areas [25], [33], Peary caribou home range sizes in other areas (no data available for Ellesmere Island) are between 100 km^2^ and 2429 km^2^, with some individuals making long movements outside their normal home ranges [28], [34], [35], and the magnitude of published pairwise F_ST_ values among contemporary northern Ellesmere Island sampling locations is relatively small (0.009–0.120; [33]). Collectively, this evidence suggests that gene flow is likely among the contemporary Ellesmere Island locations sampled in this study.

### DNA Extraction – Historic Samples

Teeth were filed to clean off surface contamination and then drilled to obtain tooth powder. Files, drill bits, and the bench were cleaned with 10% bleach and distilled water between every sample. Tooth powder (200 mg) was combined with 1 ml of 0.5 M EDTA, pH 8, and agitated for 24 h at room temperature to decalcify the sample (J. Austin pers. comm.). Samples were then centrifuged for 3 min at 10,000 rpm to pellet the tooth powder. The supernatant was drawn-off and discarded (but see [36] for a recent change in protocol). DNA was extracted from the decalcified tooth powder using the Qiagen DNEasy kit with carrier RNA and double the amounts of Buffer ATL, proteinase K, Buffer AL, and 100% ethanol. Samples were eluted twice with 200 µL Buffer AE and these eluates were combined and concentrated with a speed vacuum concentrator to approximately 100 µL. Replicate extractions (n = 2–4) were made for 35/50 historic tooth samples. Negative controls were made for all extractions. DNA extraction and amplification was carried out with new equipment in a dedicated room that had never been used for genetics work and was separate from contemporary samples and PCR product.

### DNA Extraction – Contemporary Samples

Caribou fecal pellets (n = 1) were placed in a 50 ml conical tube with 2000 µL buffer ATL from the Qiagen DNEasy kit (Qiagen, Valencia, CA) and incubated at room temperature for 1 h with the tube rotated every 5 min (D. Paetkau pers. comm.). After 1 h, the pellets were discarded and DNA was extracted from 480 µL of the ATL liquid using the Qiagen DNEasy kit and 2.5× the usual amounts of Buffer AL and 100% ethanol (D. Paetkau pers. comm.). Samples were eluted twice with 200 µL Buffer AE. Replicate extractions were made for 18/51 contemporary fecal samples together with negative controls for every extraction.

### Screening for Microsatellite Variation

DNA was genotyped at nine microsatellite loci that had small published fragment sizes: RT5, RT6, BM3413, BM4513, BMS468, BMS1788, NVHRT22, NVHRT71, and OarFCB193 [37]–[41]. Reagents and consumables used for the historic samples were treated following [42]. In brief, all tubes (clear-walled), PCR strips, water, rabbit serum albumin (RSA; used in place of bovine serum albumin to eliminate a potential source of exogenous DNA), and buffer were placed within 1 cm of UV bulbs and irradiated under UV light for 15 min. dNTPS and Qiagen Hot Star Plus polymerase were treated with heat-labile double-strand specific DNase (Biotec Marine Biochemicals, Tromsø, Norway). DNA (historic and contemporary) was amplified using 1× buffer (Qiagen), 0.8 mM dNTPs (Qiagen), 0.25 U Hot Star Plus polymerase (Qiagen), 0.05–0.5 µM primers tagged with M13 forward or reverse tails (Operon), 1 mg/ml RSA, 0.03 µM M13 Forward or Reverse IRDye 700 or 800 (Li-COR Biosciences, Lincoln, NE), and nanopure water for a total reaction volume of 10 µL. Thermocycling conditions consisted of 5 min at 95°C, followed by 5 cycles of 94°C for 30 s, 52°C for 20 s, and 72°C for 30 s, followed by 50 cycles of 94°C for 15 s, 52°C for 20 s, and 72°C for 30 s with a final extension step of 72°C for 30 min (modified from [43]). PCR reactions were replicated 1–4 times per DNA extraction (note that multiple extractions were made for some individuals – see above). After PCR, 3 µL of stop dye was added to the reactions, and following a 4 min denaturation step at 94°C, 0.8 µL of the mixture was eletrophoresed on a Li-COR 4200 automated DNA analyzer with size standard IRDyes of 50–350 bp (Li-COR Biosciences, Lincoln, NE). Microsatellite allele sizes were scored using the program Saga v 3.3.

### Screening for Mhc Variation

Historic and contemporary DNA was amplified at the Mhc *DRB* gene using LA31/32 primers developed for cattle [44], [45], which have been shown to amplify a single gene in multiple studies and subspecies of caribou [44], [46]. As above, PCR reactions of historic DNA were prepared under stringent anti-contamination conditions. Amplification reactions (1–2 per individual) consisted of 1× buffer (Qiagen), 0.8 mM dNTPs (Qiagen), 0.625 U Hot Star Plus polymerase (Qiagen), 0.5 µM primers (Operon), 1 mg/ml RSA, and nanopure water for a total reaction volume of 25 µL. Amplified PCR product was run on agarose gels and the band in the 250 bp region was excised and purified with Ultra-Sep (Omega) or QIAquick Gel Extraction (Qiagen) kits according to the manufacturer’s instructions. Purified PCR product was cycle-sequenced in both directions using 5× BigDye Buffer (ABI), BigDye v 3.1, 1.4 µM primer, 1.5 µL of PCR product, and nanopure water for a total reaction volume of 7.0 µL. Cycle-sequencing product was purified with Sephadex G-50 (SigmaAldrich) and run on an ABI 3100 Genetic Analyzer.

Most of the sequences obtained above were heterozygous, therefore DNA was re-amplified (1–2 reactions per individual) and run on agarose gels to obtain and purify the 250 bp band (as above). Purified PCR product from all amplifying individuals, including homozygotes, was cloned using the pGEM-T Easy kit with JM 109 competent cells according to the manufacturer’s instructions (Promega). Direct PCR of clones (4–10 reactions per individual using a different colony in each reaction) consisted of 1× buffer (Qiagen), 0.8 mM dNTPs (Qiagen), 0.625 U Hot Star Plus polymerase (Qiagen), 0.5 µM M13 primers, and nanopure water for a total reaction volume of 25 µL. PCR product was purified with the QIAquick PCR purification kit (Qiagen) and cycle-sequenced in both directions as above. A total of 2–4 PCR reactions were performed per individual when both the direct sequencing and cloning procedures are considered.

### Statistical Analyses – Microsatellite Data

Microsatellite genotypes were checked for scoring error, large allele dropout, and null alleles using Micro-Checker [47]. Individuals were identified as heterozygotes when they had two different alleles in the same PCR or two different alleles in separate PCRs [48]. The allelic dropout rate was estimated as the ratio of the number of observed allelic dropouts over the number of successful amplifications of heterozygous individuals following [49] (equation 2). Genotypes of fecal samples, for which individuals may be sampled multiple times, were checked for matches with GeneCap [50] to eliminate multiple sampling of the same individual. This analysis was not necessary for historic samples because each tooth was taken from a different skull.

Hardy-Weinberg Equilibrium, F_IS_, linkage disequilibrium, allelic richness, and heterozygosity, were calculated with FStat v. 2.9.3.2 [51], Genetix v. 4.05 [52], and Genepop v. 4.1 [53]. The mean number of rare alleles (frequency ≤5%) was calculated for each time period by dividing the total number of rare alleles by the number of loci. Significant differences in genetic diversity between the historic and contemporary microsatellite data were examined using Wilcoxon signed ranks tests (α = 0.05), which allows for tests paired for loci [18].

### Statistical Analyses – Mhc Data

Mhc sequences were edited, aligned and compared using Sequencher 5.0 (Gene Codes Corporation) and previously identified *Rangifer tarandus DRB* alleles [44], [46] (Djakovic et al. unpubl. sequences, GenBank; Wei & Happ unpubl. sequences, GenBank). Within an individual, sequences from clones were compared to the heterozygous direct sequences obtained without cloning to ensure that cloned alleles were consistent with heterozygous sequences. Individuals were assigned an Mhc genotype based on the alleles observed in each individual. To estimate synonymous and non-synonymous nucleotide changes, the reading frame was first determined by translating the consensus *DRB* gene sequence, starting at the first, second, and third base, to amino acid sequences, which were examined for stop codons and similarity to conserved regions in GenBank. We used the nucleotide sequence starting at the first base of our consensus sequence to create the protein translation because this was the only sequence that did not produce stop codons and aligned with the conserved protein domain of the Mhc II beta superfamily in GenBank (sequences beginning with the second or third base produced stop codons and did not align). Non-synonymous (*d*_N_) and synonymous (*d*_S_) nucleotide changes were calculated in Mega v. 5.05 [54] using 8000 bootstrap replications, and a Z-test of selection was implemented to test whether *d*_N_ > *d*_S_ using the Nei-Gojobori method with Jukes-Cantor correction. Average codon *d*_N_ and *d*_S_ values were calculated by estimating selection for each codon. Gene diversity at Mhc loci may be influenced by gene conversion and recombination [55], therefore we examined caribou Mhc sequences for each type of event. Gene conversion was assessed with Geneconv v. 1.81 [56], which searches for nucleotide fragments in a pair of sequences that are more similar to each other than expected by chance. Gene conversion events were analyzed by examining synonymous sites of coding regions only (to avoid the potential effects of selection) as well as all sites. Gene conversion events were considered significant when the simulated global p-value was less than 0.05 (permutation runs  = 10,000). The minimum number of recombination events was examined with DnaSP [57]. Nucleotide diversity and haplotype diversity were calculated using DnaSP [57]. Allelic richness, heterozygosity, and rare alleles were calculated as above.

### Mhc Nomenclature

We amplified a 252 bp fragment of the *DRB* gene, a slightly longer fragment than that reported by [44] (9 sequences, 249 bp), Wei & Happ (GenBank; 12 sequences, 234 bp), and Djakovic et al. (GenBank; 6 sequences, 249 bp), and an equal length to that published by [46] (11 sequences). By convention, the full exon must be sequenced to assign allele numbers, however only 10 bases are missing at each end and these are not variable in other species [46]. Furthermore, alleles have already been numbered by [44], [46], therefore we continue the tradition here. Alleles observed in more than three individuals with more than four amino acid differences were given a new number in the series (e.g. 1101). Those with three or four amino acid differences were given a new subtype number (e.g. 0201, 0202, 0203). Alleles and subtypes observed in three or fewer individuals were given local names (e.g. ak21). Any alleles that differed by one or two base pairs were considered to be variants (e.g. 0902 v). Degraded DNA can often be damaged, which causes sequencing errors, therefore we analyzed the data by using all unique sequences (full dataset) and by considering all variants of an allele to be a single allele (variants combined).

### Statistical Analyses – All Data

Observed loss of expected heterozygosity and allelic richness was compared to theoretical expectations using the current Peary caribou population size estimate of 802 for northern Ellesmere Island [26], 14 generations (using the sampling dates of 1905 and 2006, and assuming that generation time is approximately 7 years [27]), and the theoretical equations H_t_  = (1–1/2N)^t^ H_0_ (where N is the number of individuals, t is the number of generations, and H_0_ is the historic expected heterozygosity) and E(A’) = A − ∑ (1– p)^2N^ (where E(A’) is the expected number of alleles, A is the historic number of alleles, p is the allele frequency of each allele at a locus, and N is the number of individuals).

## Results

### Microsatellite Data

All contemporary fecal samples amplified (n = 39/51 to 51/51 depending on the locus) and repeat extractions and PCRs generally produced consistent genotypes. The estimated allelic dropout rate was 8.3% and there was no evidence of contamination in negative controls. Three pairs of fecal samples appeared to have identical genotypes (sibling probability of identity <0.040), therefore the genotype with the least data from each pair was dropped from the analysis.

Amplification success in the older historic tooth samples was lower than the contemporary samples, as expected: 31/50–44/50 samples amplified (depending on the locus), repeat extractions and PCRs did not always produce identical genotypes, and estimated allelic dropout rate was 38.0%. There was no evidence of contamination in any of the negative controls.

Both contemporary and historic samples showed an excess of homozygotes at 5/9 and 7/9 loci respectively (Micro-Checker using Bonferroni confidence intervals) and high levels of inbreeding, with a higher F_IS_ value observed in the historic versus the contemporary sample (Table 1). To reduce the effect of allelic dropout, we re-did the Micro-Checker analysis with a reduced set of genotypes from high quality DNA that reliably sequenced at the Mhc *DRB* gene (historic n = 11; contemporary n = 24). This reduced the number of loci showing an excess of homozygotes in the historic sample to two loci, but it made no difference to the contemporary sample in either the number or identity of loci with a homozygote excess. Similarly, F_IS_ in the historic sample decreased to the same approximate level as the contemporary sample (∼0.3; Table 1). In contrast, the contemporary samples indicated a similar level of F_IS_ over time regardless of whether all data or only high quality DNA were used (Table 1). To test for a Wahlund effect in the contemporary sample, which covered a larger geographic area than the historic sample, we deleted individuals from the NW area of Ellesmere Island, the most differentiated population from other northern Ellesmere Island populations (published F_ST_ values  = 0.023–0.12; [33]) but 4/9 loci still showed an excess of homozygotes.

**Table 1:** Genetic diversity for microsatellite and Mhc data in historic and contemporary samples.

		Historic	Contemporary	% variation remaining
Microsatellite data	All DNA	n = 50	n = 51	
	Mean no. of alleles/locus	6.556	5.000	
	AR	6.441	4.817	74.8
	Ho	0.362	0.413	114.1
	He	0.685	0.586	85.5
	Fis	0.481	0.298	
	Mean number of rare alleles	2.000	1.556	
				
	High quality DNA	n = 11	n = 24	
	Mean no. of alleles/locus	4.889	4.556	
	AR	4.603	3.661	79.5
	Ho	0.508	0.412	81.1
	He	0.693	0.584	84.3
	Fis	0.267	0.299	
	Mean number of rare alleles	0.889	1.111	
				
Mhc data	All alleles	n = 11	n = 24	
	No. of alleles	10	15	
	AR	10.000	9.444	94.4
	Ho	0.636	0.792	124.5
	He	0.896	0.825	92.1
	Number of rare alleles	5	11	
	Pi	0.056	0.047	83.9
	Haplotype diversity	0.896	0.825	92.1
	No. of segregating sites	37	37	
				
	Variants combined	n = 11	n = 24	
	No. of alleles	8	8	
	AR	8.000	6.000	75.0
	Ho	0.546	0.792	145.1
	He	0.836	0.740	88.5
	Number of rare alleles	4	3	
	Pi	0.055	0.047	85.5
	Haplotype diversity	0.836	0.740	88.5
	No. of segregating sites	35	35	
				

AR  =  allelic richness, Ho  =  observed heterozygosity, He  =  expected heterozygosity.

Pi (nucleotide diversity) calculated with Jukes-Cantor correction.

Linkage disequilibrium was observed for seven locus pairs in the full dataset and two locus pairs in the high quality DNA dataset. When the historic and contemporary samples were considered separately, no pair was linked in both samples. Therefore, all loci were kept for all analyses.

Mean allelic richness (AR) and expected heterozygosity (H_E_) were significantly higher in the historical sample than in the contemporary sample for both datasets and all tests (Table 1; All DNA: AR Z-statistic  = −2.100, p = 0.036; H_E_ Z-statistic  = −2.192, p = 0.028; High quality DNA: AR Z-statistic  = −2.100, p = 0.036; H_E_ Z-statistic  = −2.310, p = 0.021) suggesting a loss of microsatellite genetic diversity over the past century. There was no significant difference in mean observed heterozygosity (H_O_) between the contemporary and historic samples (All DNA: H_O_ Z-statistic  = 1.244, p = 0.214; High quality DNA: H_O_ Z-statistic  = −1.125, p = 0.26). More rare alleles were present in the historic versus the contemporary sample when all data were used, but the reverse was true when the high quality DNA dataset was used (Table 1).

### Mhc Data

We successfully sequenced 35 individuals (historic n = 11; contemporary n = 24). A maximum of two alleles was amplified in a single individual and identical replicate sequences were produced for each individual excluding occasional base errors, which were corrected using replicate sequences and a majority rule. We found five previously identified alleles and five variants of previously identified alleles (Table 2 & 3). We also discovered one new allele (1101; present in 18 individuals and different from 0201 by 7 bp and 5 amino acid differences; Table 3), and one new subtype (0202; present in 9 individuals and different to 0201 by 3 amino acid differences; Table 3). One additional new subtype (ak21 and different from 0701 by 3 bp and 3 amino acid differences) and seven variants of new alleles were present in one individual only. Table 2 shows the allele identities and frequencies in the historic and contemporary samples for all alleles and when variants of alleles were combined.

**Table 2:** *DRB* allele frequencies for all alleles and variants combined in the historic and contemporary samples.

	Historic	Contemporary	GenBank Accession No.
All alleles			
0201	0.046	0	AF012719
0201v1	0	0.021	AF012719 variant
0202	0.182	0.146	New
0202v1	0	0.042	New
0202v2	0.091	0	New
0202v3	0.046	0	New
0202v4	0	0.021	New
0202v5	0	0.021	New
0301	0.091	0.083	AF012720
0601	0.046	0.125	AF012723
0601v1	0	0.021	AF012723 variant
0902v1/HQ245651v1	0.046	0	HQ245651/Kennedy *et al*. 2010
1101	0.227	0.375	New
1101v1	0	0.042	New
1101v2	0	0.021	New
HQ245646	0.182	0.021	HQ245646
AK01v1	0	0.021	AF458939 variant
AK01v2	0	0.021	AF458939 variant
AK12	0	0.021	AF458950
AK21	0.046	0	New
Variants combined			
0201	0.046	0.021	AF012719
0202	0.318	0.229	New
0301	0.091	0.083	AF012720
0601	0.046	0.146	AF012723
0902v1/HQ245651v1	0.046	0	HQ245651/Kennedy *et al*. 2010
1101	0.227	0.438	New
HQ245646	0.182	0.021	HQ245646
AK01v1	0	0.042	AF458939 variant
AK12	0	0.021	AF458950
AK21	0.046	0	New

Allele	Amino acid sequence																																																												
0201 AF012719	L	E	Y	F	K	G	E	C	H	F	S	N	G	T	E	R	V	Q	F	L	K	R	Y	I	H	N	G	E	E	S	V	R	F	D	S	D	V	G	E	F	R	A	V	T	E	L	G	R	P	D	A	E	V	W	N	S	R	K	E	I	L	E	E	S	R	A	E	V	D	T	V	C	R	H	N	Y	G	V	L	D	S	F	I	V
0201v1	.	.	.	.	.	.	.	.	.	.	.	.	.	.	.	.	.	.	.	.	.	.	.	.	.	.	.	.	.	.	.	.	.	.	.	.	.	.	.	.	.	.	.	.	.	.	.	.	.	V	.	.	.	.	.	.	.	.	.	.	.	.	.	.	.	.	.	.	.	.	.	.	.	.	.	.	.	.	.	.	.	.	.	.
0202	.	.	.	.	.	C	.	.	.	.	.	.	.	.	.	.	.	.	.	.	.	.	.	.	.	.	.	.	.	L	.	.	.	.	.	.	.	.	.	.	.	.	.	.	.	.	.	.	.	.	.	.	.	.	.	.	.	.	.	.	.	.	.	.	.	.	.	.	.	.	.	.	.	.	.	.	.	.	.	.	.	.	T	.
0202v1	.	.	.	.	.	C	.	.	.	.	.	.	.	.	.	.	.	.	.	.	.	.	.	.	.	.	.	.	.	L	.	.	.	.	.	.	.	.	.	.	.	.	.	.	.	.	.	.	.	.	.	.	.	.	.	.	.	.	.	.	.	.	.	.	.	.	.	.	.	.	.	.	.	.	.	.	.	.	.	.	.	.	.	.
0202v2	.	.	.	.	.	C	.	.	.	.	.	.	.	.	.	.	.	.	.	.	.	.	.	.	.	.	.	.	.	L	.	.	.	.	.	.	.	.	.	.	.	.	.	.	.	.	.	.	.	.	.	.	.	.	.	.	.	.	.	.	.	.	.	.	.	.	.	.	.	.	.	.	.	.	.	.	.	.	.	.	.	.	T	.
0202v3	.	.	.	.	.	C	.	.	.	.	.	.	.	.	.	.	.	.	.	.	.	.	.	.	.	.	.	.	.	L	.	H	.	.	.	.	.	.	.	.	.	.	.	.	.	.	.	.	.	.	.	.	.	.	.	.	.	.	.	.	.	.	.	.	.	.	.	.	.	.	.	.	.	.	.	.	.	.	.	.	.	.	T	.
0202v4	.	.	.	V	.	.	.	.	.	.	.	.	.	.	.	.	.	.	.	.	.	.	.	.	.	.	.	.	.	L	.	.	.	.	.	.	.	.	.	.	.	.	.	.	.	.	.	.	.	.	.	.	.	.	.	.	.	.	.	.	.	.	.	.	.	.	.	.	.	.	.	.	.	.	.	.	.	.	.	.	.	.	T	.
0202v5	.	.	.	.	.	C	.	.	.	.	.	.	.	.	.	.	.	.	.	.	.	.	.	.	.	.	.	.	.	L	.	.	.	.	.	.	.	.	.	.	.	.	.	.	.	.	.	.	.	.	.	.	.	.	.	.	.	.	.	.	.	.	.	.	.	.	.	.	.	.	.	.	.	.	.	.	.	.	.	.	.	.	T	.
0301 AF012720	.	.	.	V	.	S	.	.	.	.	.	.	.	.	Q	G	.	.	.	.	Q	.	H	.	Y	.	.	.	.	F	L	.	.	.	.	.	.	.	.	.	.	.	.	.	.	.	.	.	.	.	.	.	G	.	.	.	.	.	D	.	.	.	D	K	.	.	A	.	.	.	Y	.	.	.	.	.	.	.	.	.	.	.	T	.
0601 AF012723	.	.	.	V	.	.	.	.	.	.	.	.	.	.	.	.	.	.	L	.	Q	.	F	.	Y	.	.	.	.	F	.	.	.	.	.	.	.	.	.	.	.	.	.	.	.	.	.	.	.	.	.	.	.	.	.	.	Q	.	.	.	.	.	.	.	.	.	A	.	.	.	Y	.	.	.	.	.	.	.	G	E	.	.	T	.
0601v1	.	.	.	V	.	.	.	.	.	.	.	.	.	.	.	.	.	.	L	.	Q	.	F	.	Y	.	R	.	.	F	.	.	.	.	.	.	.	.	.	.	.	.	.	.	.	.	.	.	.	.	.	.	.	.	.	.	Q	.	.	.	.	.	.	.	.	.	A	.	.	.	Y	.	.	.	.	.	.	.	G	E	.	.	T	.
0902v1 HQ245651	.	.	.	.	.	C	.	.	.	.	.	.	.	.	.	.	.	.	.	.	.	.	.	.	.	.	.	.	.	L	.	.	.	.	.	.	.	.	.	.	.	.	.	.	.	.	.	.	.	.	.	.	.	.	.	.	.	.	.	.	.	.	.	.	.	.	.	.	.	.	.	.	.	.	.	.	.	.	G	E	.	.	T	.
1101	.	.	.	V	.	S	.	.	.	.	.	.	.	.	.	.	.	.	.	.	N	.	.	.	.	.	.	.	.	F	.	.	.	.	.	.	.	.	.	.	.	.	.	.	.	.	.	.	.	V	.	.	.	.	.	.	.	.	.	.	.	.	.	.	.	.	.	.	.	.	.	.	.	.	.	.	.	.	.	.	.	.	.	.
1101v1	.	.	.	V	.	S	.	.	.	.	.	.	.	.	.	.	.	.	.	.	N	.	.	.	.	.	.	.	.	F	.	.	.	.	.	.	.	.	.	.	.	.	.	.	.	.	.	.	.	V	.	.	.	.	.	.	.	.	.	.	.	.	.	.	.	.	.	.	.	.	.	.	.	.	.	.	.	.	.	.	.	.	T	.
1101v2	.	.	.	.	.	S	.	.	.	.	.	.	.	.	.	.	.	.	.	.	N	.	.	.	.	.	.	.	.	F	.	.	.	.	.	.	.	.	.	.	.	.	.	.	.	.	.	.	.	V	.	.	.	.	.	.	.	.	.	.	.	.	.	.	.	.	.	.	.	.	.	.	.	.	.	.	.	.	.	.	.	.	.	.
HQ245646	.	.	.	V	.	S	.	.	.	.	.	.	.	.	Q	G	.	.	.	.	Q	.	.	.	Y	.	.	.	.	Y	.	.	.	.	.	.	.	.	.	.	.	.	.	.	.	.	.	.	K	S	.	.	G	.	.	.	Q	.	.	.	.	.	D	K	.	.	A	.	.	.	.	.	.	.	.	.	.	.	G	E	.	.	T	.
AK01v1 AF458939	.	.	.	V	.	S	.	.	.	.	.	.	.	.	Q	G	.	.	.	.	Q	.	.	.	Y	.	.	.	.	Y	.	.	.	.	.	.	.	.	.	.	.	.	.	.	.	.	.	.	K	S	.	.	G	.	.	.	Q	.	.	.	.	.	D	K	.	.	A	.	.	.	.	.	.	.	.	.	.	.	R	.	.	.	.	.
AK01v2 AF458939	.	.	.	V	.	S	.	.	.	.	.	.	.	.	Q	G	.	.	.	.	Q	.	.	.	Y	.	.	.	.	Y	.	.	.	.	.	.	.	.	.	.	.	.	.	.	.	.	.	.	K	S	.	.	G	.	.	.	Q	.	.	.	.	.	D	K	.	.	A	.	.	.	.	.	.	.	.	.	.	.	R	E	.	.	T	.
AK12 AF458950	.	.	.	V	.	S	.	.	.	.	.	.	.	.	.	.	.	.	.	.	.	.	.	.	.	.	.	.	.	L	.	.	.	.	.	.	.	.	.	.	.	.	.	.	.	.	.	.	.	V	.	.	G	.	.	.	.	.	D	.	.	.	.	K	.	.	A	.	.	.	Y	.	.	.	.	.	.	.	G	E	.	.	T	.
AK21	.	.	.	.	.	C	.	.	.	.	.	.	.	.	.	.	.	.	.	.	.	.	.	.	.	.	.	.	.	L	.	.	.	.	.	.	.	.	.	.	.	.	.	.	.	.	.	.	.	.	.	.	G	.	.	.	.	.	D	.	.	.	D	K	.	.	A	.	.	.	Y	.	.	.	.	.	.	.	.	.	.	.	T	.

**Table 3:**
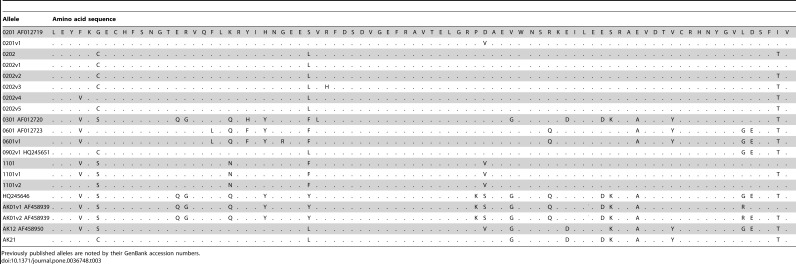
Amino acid sequences for Peary caribou *DRB* alleles.

Nucleotide changes (*d*_N_/*d*_S_) were significantly different from the neutral expectation and indicated positive selection when all sequences were used (historic: test statistic  = 2.144, p = 0.016, mean codon *d*_S_ = 0.143, *d*_N_ = 0.293; contemporary: test statistic  = 1.904, p = 0.029, mean codon *d*_S_ = 0.159, *d*_N_ = 0.309; and when allele variants were combined (historic: test statistic  = 2.349, p = 0.009, mean codon *d*_S_ = 0.112, *d*_N_ = 0.287; contemporary: test statistic  = 1.947, p = 0.026, mean codon *d*_S_ = 0.154, *d*_N_ = 0.258). Gene conversion was not detected when only silent substitutions were examined (n = 20 sequences, max. score  = 0.893, p = 1), but was significant for 13 fragments when all sites were examined (n = 20 sequences, max. score  = 7.741, p<0.0001). Sequences showing evidence for gene conversion were variants or subtypes with local names (0202, 0202v1–5, HQ245651v1, AK12, AK21). Analyses in DnaSP found a minimum of 8 recombination events.

Mean allelic richness, expected heterozygosity, and nucleotide and haplotype diversity were all higher in the historical than contemporary sample for both Mhc datasets (Table 1), suggesting a loss of Mhc diversity over the past century. Mean observed heterozygosity was higher in the contemporary sample than in the historic sample when all sequences were used, but the reverse was true when allele variants were combined (Table 1). The Mhc gene had more rare alleles as compared to the mean number of rare alleles observed in microsatellite loci, but we found no clear pattern to suggest that a greater proportion of rare alleles was lost over time at Mhc versus microsatellite loci (Table 1).

### All Data

Our most conservative analyses used high quality DNA (microsatellite and Mhc loci) and combined the allele variants (Mhc loci) to show that change in variation over time was similar for microsatellite (79.5% AR retained; 84.3% H_E_ retained) and Mhc loci (75.0% AR retained; 88.5% H_E_ retained). These data also suggest that slightly more rare Mhc alleles were present in historic (n = 4) versus contemporary samples (n = 3) as opposed to microsatellite loci (historic sample  = 0.889 alleles; contemporary sample  = 1.111 alleles). However, this apparent difference does not necessarily indicate that rare Mhc alleles present historically were lost in the contemporary sample. Instead, two different sets of rare alleles appear to have existed at each time period (Table 2), perhaps explained by gene conversion, recombination and/or selection. These processes may also explain the pattern observed in the full datasets (all DNA and all Mhc sequences), which indicate an increase in rare Mhc alleles over time (Table 1). The current small population size of Peary caribou on northern Ellesmere Island (n = 802; [26]) and our theoretical estimates (see above) suggest that current levels of expected heterozygosity and allelic richness should be virtually identical to historic levels, even though our results indicate a loss of variation over time. A considerably smaller, current population size of 41 would be consistent with current levels of expected heterozygosity.

## Discussion

Research on the loss of genetic variation in declining populations suggests that functional Mhc genes may either maintain or lose variation at different rates than neutral loci, and thus estimates of variation based on Mhc loci may be more useful than those based on neutral loci to managers wanting to assess the adaptive potential and viability of endangered species, especially with respect to disease [58], [59]. A recent meta-analysis suggests that Mhc genes typically lose ∼15% more variation than neutral loci during population declines [15], perhaps due to a high number of rare alleles lost during bottlenecks. Other studies suggest that Mhc loci may maintain genetic variation during bottlenecks if balancing selection is sufficient to over-ride the effects of genetic drift [12].

However, the overwhelming majority of studies to date have compared genetic variation in small, bottlenecked populations with large populations (reviewed in [15]), despite the potential for differences in colonization history, disease prevalence and population size, which may all affect genetic variation over time. We directly compared the loss of variation at neutral and functional loci in a single population over time to mitigate the potentially confounding effect of population history. Our most conservative analyses (high quality DNA and Mhc variants combined) indicate that Mhc and microsatellite loci in Peary caribou lost a similar proportion of genetic variation over time. This suggests that genetic drift affected variation at Mhc and microsatellite loci about equally even though the Mhc gene appeared to be under strong balancing selection (*d*_N_ > *d*_S_). In sum, our results are therefore consistent with the suggestion that microsatellite loci can be used to reliably estimate genome-wide levels of genetic variation, and that the negative effect of genetic drift on genetic variation is not always mitigated by balancing selection or exacerbated by Mhc allele distributions.

It is important to note that our results may also be influenced by undetected historical events, and that a more detailed knowledge of population history is needed to fully characterize the effects of drift and selection on neutral and functional alleles in Peary Caribou. In particular, a number of species including wolves, muskoxen and caribou appear to have colonized Arctic regions following the last glaciation [60]–[62]. If a relatively small number of Peary caribou colonized Ellesmere Island, this might limit genetic variation in historic populations as well as the number of rare alleles. Population size in Peary caribou may have also fluctuated over time given that survival varies in relation to food availability and weather [63], [64]. A small founder population subject to serial bottlenecks might be expected to have lost many rare Mhc alleles prior to the collection of our historic samples, thus reducing our ability to detect high rates of loss of rare Mhc alleles and/or higher rates of loss variation at Mhc than microsatellite loci (*cf* [15]). Historic bottlenecks in Peary caribou followed by population recovery may also explain why inbreeding values are high, and why current levels of observed variation are considerably lower than those expected by theory and current population size.

We detected gene conversion and recombination at the Peary caribou *DRB* gene; two mechanisms that could influence Mhc diversity following bottlenecks. Although it is not possible to disentangle the effects of selection and gene conversion at non-synonymous sites, where we detected gene conversion, it is plausible that gene conversion at non-synonymous sites produces new alleles. This may explain our observation of different sets of rare alleles in historic and contemporary samples as well as the high levels of polymorphism generally observed at Mhc loci as noted by others [55], [65]–[67].

A large homozygote excess in our historic and contemporary microsatellite data preclude analyses that assume Hardy-Weinberg Equilibrium and may be due to allelic dropout, a Wahlund Effect, population history or inbreeding. However, the homozygote excess does not appear to be attributable to allelic dropout for three reasons: 1) it was observed in samples for which there was sufficient, high quality DNA to sequence 252 bp amplicons, which indicates that smaller microsatellite amplicons should have amplified successfully; 2) more microsatellite alleles were observed in the historic than the contemporary sample even though allelic dropout is more likely in the historic sample, and; 3) multiple PCR reactions and clones produced consistent genotypes and Mhc sequences that exactly matched published alleles (Table 2). A Wahlund effect (contemporary samples) also appears to be unlikely because the magnitude of published F_ST_ values among northern Ellesmere Island populations is relatively small [33] and dropping the most differentiated population had little effect on our results. Petersen *et al*. [33] also noted evidence for a homozygote excess at some microsatellite loci (2–4 loci depending on the analysis), but estimated a low F_IS_ value. In addition to the high F_IS_ values estimated here, we also note that two *DRB* alleles (0202 and 1101) had high frequencies, also suggesting high relatedness. A high frequency of one or two *DRB* alleles in other ungulate populations appears to be normal, but the most common alleles in non-bottlenecked populations tend to have lower allele frequencies than the most common alleles in small or bottlenecked populations [9], [46], [68].

Inbreeding in the historic and contemporary samples might be expected given the caribou’s polygamous breeding system and/or historic bottlenecks. Peary caribou may be susceptible to inbreeding because they occur at low density [26]. If reproduction within groups is highly skewed [69]–[71], or if group members seldom encounter other potential mates [70], [72], inbreeding may also be likely. Interestingly, experimental studies of *Drosophila* harems have produced inbreeding values similar to those we report for Peary caribou [71] and to values observed in Sable Island horses (population subdivision II F_IS_ = 0.113) [73] and Arctic wolves (F_IS_ = 0.629) [60]. However, F_IS_ values in most free-living ungulates are low [74]–[77] and the Peary caribou breeding system is not sufficiently well-studied [33] to draw clear conclusions.

### Implications for Conservation

In Ellesmere Island Peary caribou, the observed loss of variation over time suggests a decreased ability to adapt to environmental change and an increased susceptibility to inbreeding depression, which may act to reduce reproductive success and survival [78]. Although direct comparisons with other studies are not possible given the different panels of markers used, levels of microsatellite allelic richness and expected heterozygosity observed here agree well with those reported by Petersen et al. [33] on Ellesmere Island, are lower than those detected in other Peary caribou populations [43], [79], and are similar to levels observed on Somerset/Prince of Wales Islands [43], a population that may now be extinct [24], [26]. Collectively, these findings indicate that genetic variation in Peary caribou on Ellesmere Island is low.

More generally, our results suggest that genetic drift appears to have similar negative effects on genetic variation at both microsatellite and Mhc loci. This suggests that microsatellite loci can be used to estimate genome-wide levels of variation, but that adaptive potential may be lost when bottlenecks occur, and that a compensatory effect by balancing selection on Mhc genes cannot be assumed. On a positive note, gene conversion and recombination may act to increase variation at Mhc genes following bottlenecks.

## Supporting Information

Appendix S1 **Historic sample information from the American Museum of Natural History (AMNH).**
(DOCX)
