# Supplementary material for: Loss of Mhc and Neutral Variation in Peary Caribou: Genetic Drift Is Not Mitigated by Balancing Selection or Exacerbated by Mhc Allele Distributions
Source: PLoS One. 2012 May 24;7(5):e36748. doi: 10.1371/journal.pone.0036748 (PMC3360046; doi:10.1371/journal.pone.0036748)
Supplement: Appendix S1 — Historic sample information from the American Museum of Natural History (AMNH). (DOCX) [file pone.0036748.s001.docx]

| Appendix S1. Historic sample information from the American Museum of Natural History (AMNH). | | |
| --- | --- | --- |
| AMNH accession number | Area collected | Date |
| **27901** | NE of Lake Hazen | 1905 |
| 27902 | NE of Lake Hazen | 1905 |
| 27904 | NE of Lake Hazen | 1905 |
| 27905 | NE of Lake Hazen | 1905 |
| 27906 | NE of Lake Hazen | 1905 |
| 27913 | Lake Hazen | 1905 |
| **27914** | Lake Hazen | 1905 |
| 27916 | E of Lake Hazen | 1905 |
| 27917 | Lake Hazen | 1905 |
| 27918 | Lake Hazen | 1905 |
| **27920** | Lake Hazen | 1905 |
| **27921** | Lake Hazen | 1905 |
| 27925 | Lake Hazen | 1905 |
| 27928 | NE of Lake Hazen | 1905 |
| 27935 | NE of Lake Hazen | 1905 |
| **27939** | Lake Hazen | 1905 |
| 27941 | Lake Hazen | 1905 |
| 27946 | Lake Hazen | 1905 |
| 27948 | Lake Hazen | 1905 |
| 27958 | Black Cliffs Bay | 1905 |
| 27987 | Lake Hazen | 1905 |
| **27989** | Lake Hazen | 1905 |
| **27990** | Black Cliffs Bay | 1905 |
| 29982 | Between Black Cliffs Bay and 50 miles south | 1909 |
| 29983 | Between Black Cliffs Bay and 50 miles south | 1909 |
| 29985 | Between Black Cliffs Bay and 50 miles south | 1909 |
| 29995 | Black Cliffs Bay | 1908 |
| 29998 | Porter Bay | 1908 |
| 29999 | Porter Bay | 1908 |
| 30000 | Porter Bay | 1908 |
| 30001 | Porter Bay | 1908 |
| 30002 | Porter Bay | 1908 |
| **30007** | Porter Bay | 1908 |
| **30008** | Porter Bay | 1908 |
| 30011 | Porter Bay | 1908 |
| 30013 | Porter Bay | 1908 |
| **30014** | Porter Bay | 1908 |
| 30015 | Porter Bay | 1908 |
| 30020 | Porter Bay | 1908 |
| **30022** | Lake Hazen | 1908 |
| 30023 | Lake Hazen | 1908 |
| 30024 | Lake Hazen | 1908 |
| 30025 | Lake Hazen | 1908 |
| 30032 | Black Cliffs Bay | 1909 |
| 30033 | Black Cliffs Bay | 1909 |
| 30034 | Black Cliffs Bay | 1909 |
| 30035 | Black Cliffs Bay | 1909 |
| 30037 | Black Cliffs Bay | 1909 |
| 30039 | Black Cliffs Bay | 1909 |
| 30041 | Black Cliffs Bay | 1909 |
| Individuals in bold were sequenced at the *DRB* gene and were considered to have high quality DNA. | | |
